# The Relationship Between Lower Respiratory Tract Microbiome and Allergic Respiratory Tract Diseases in Children

**DOI:** 10.3389/fmicb.2021.630345

**Published:** 2021-05-14

**Authors:** Jinghua Cui, Yuanyuan Zhang, Hanqing Zhao, Xuemei Sun, Zhen Chen, Qun Zhang, Chao Yan, Guanhua Xue, Shaoli Li, Yanling Feng, Han Liu, Xianghui Xie, Jing Yuan

**Affiliations:** ^1^Capital Institute of Pediatrics, Beijing, China; ^2^Beijing Key Laboratory of Emerging Infectious Diseases, Institute of Infectious Diseases, Beijing Ditan Hospital, Capital Medical University, Beijing, China; ^3^Dongfeng Traditional Chinese Medicine Hospital, Jilin, China; ^4^Baicheng Medical College, Jilin, China

**Keywords:** lower respiratory tract, microbiome, allergic respiratory tract diseases, children, bronchoalveolar lavage fluid

## Abstract

Similar to those in the upper respiratory tract, there are microbes present in the healthy human lower respiratory tract (LRT), including the lungs and bronchus. To evaluate the relationship between LRT microbiome and allergic respiratory diseases in children, we enrolled 68 children who underwent bronchoscopy from January 2018 to December 2018 in the affiliated hospital of the Capital Institute of Pediatrics. Using the total IgE (TIgE) values, children were divided into two groups: allergy sensitivity (AS) group and non-allergy sensitivity (NAS) group. Nucleic acid was extracted from samples of bronchoalveolar lavage fluid (BALF) from the two groups of children taken during bronchoscopy treatment and the 16S rDNA gene was sequenced and analyzed. The results showed that *Haemophilus*, *Moraxella*, *Streptococcus*, *Prevotella*, *Neisseria*, and *Rothia* were detected in all patients. There was a statistically significant difference in the composition and distribution of microbiota between the AS and NAS groups (*p* < 0.01). Analysis of the correlation of clinical indices and microbiome showed that TIgE was positively correlated with *Bacteroidetes* and negatively correlated with *Streptococcus*. Absolute lymphocyte count showed a relationship with *Streptococcus*, and the absolute neutrophil count or percentage of neutrophils showed a relationship with *Cardiobacterium*. The LRT microbiome functioned similarly to the intestinal microbiome. That is, the decrease in microbial diversity and the change in composition could lead to an increase in allergic symptoms. The microbiome of the LRT in children, especially that of *Bacteriodetes* and *Streptococcus*, showed a correlation with respiratory allergic diseases.

## Introduction

At present, the prevalence rate of allergic rhinitis in mainland China is 4–38% (Zhang and Zhang, 2014). During the past few decades, the prevalence of allergic respiratory diseases in children, especially asthma caused by allergies, has been increasing year by year in China (Sha et al., 2016). This rapid increase in atopic diseases can be explained by the “hygiene hypothesis,” which suggests that low infection rates in developed countries lead to inappropriate immune activation of immunoglobulin E (IgE) against the common environment and autoantigens (Anvari et al., 2019). This hypothesis may account for the recent increase observed in food allergies, especially among children (Pascal et al., 2018). The prevalence of allergic respiratory diseases in children has been on the rise as well, and it is easily confused with inflammation caused by pathogenic infections; therefore, it is important to distinguish the characteristics of the disease. Total IgE (TIgE) and specific IgE (SIgE) are still important indicators for diagnosis of suspected allergic diseases. IgE is an immunoglobulin that regulates response to various allergens, and is mainly synthesized by B cells in the lymphatic tissues of the respiratory and digestive mucosa lamina propria and mediates type I hypersensitivity reactions that cause diseases such as allergic rhinitis, allergic reactions, and asthma (Salo et al., 2011). Previous reports have demonstrated a quantitative relationship between serum IgE and various allergic diseases (Testa et al., 2020).

A healthy and diverse gut microbiome can block the development of IgE-mediated food sensitivity (Molloy et al., 2013; Plunkett and Nagler, 2017) and a decrease in intestinal microbial diversity and changes in composition can lead to an increase in allergy symptoms (Fujimura and Lynch, 2015). Similarly, some changes in the pulmonary microbiota of asthma patients have been noted, when compared with that in normal people, opening the door to the concept that dysbacteriosis of pulmonary microbiome may play a role in the pathogenesis of chronic airway disease (Hilty et al., 2010; Erb-Downward et al., 2011). It has been reported that in the first year after birth, infants have *Haemophilus*, *Streptococcus*, *Moraxella*, *Staphylococcus*, *Clostridium*, and *Corynebacterium* in the nasopharynx, which are associated with a higher risk of asthma (Teo et al., 2015). Analysis of the microbiome from nasopharyngeal swabs and bronchoalveolar lavage fluid (BALF) demonstrated that nasopharyngeal swab samples can be used to distinguish the differences in individuality among children, but the relative abundance of the microbiome in the nasopharyngeal and lower respiratory tract (LRT) was different (Kloepfer et al., 2018). Furthermore, the characteristics of the respiratory tract microbiome and its relationship with allergic disease are not clear. In children, bacterial colonization and early allergic sensitization are associated with wheezing, which is characteristic of the asthma phenotype (Teo et al., 2018). It is now well established that the healthy bronchial tree contains a microbiome distinct from that of the upper respiratory tract and that the lung microbiome may be dysregulated in individuals with a respiratory disease (Hilty et al., 2010). Therefore, analysis of respiratory microbiome components may contribute to early detection and intervention in high-risk children, thus aiding in the prevention of allergic disease.

## Materials and Methods

### Patient Selection

We selected 68 children admitted to the respiratory department of the Children’s hospital, the Capital Institute of Pediatrics, Beijing, from January 2018 to December 2018. All children underwent bronchoscopy and were classified into two groups, the allergy sensitivity group (AS) and the non-allergy sensitivity group (NAS), and there were 34 children in each group. The patients in the AS group had a total IgE > 60 IU/mL and a diagnosis of pneumonia or bronchopneumonia with or without respiratory allergic symptoms, such as asthma and rhinitis. The NAS group had presented with a total IgE ≤ 60 IU/mL and a diagnosis pneumonia or bronchopneumonia, without allergic symptoms. Patients were excluded based on the following criteria: (1) diagnosis of pneumonia with specific pathogen infection such as fungus, virus, or mycoplasma or (2) history of mechanical ventilation. Informed consent from all participants has been obtained. Most of the cases in this study had been treated with antibiotics before performing fibreoptic bronchoscopies, and the antibiotics using histories were not clear. Trial was registered retrospectively. The number trial registration is ISRCTN18302701, and date of registration is 06/10/2020.

### Biomarkers Test

Total IgE levels were detected using a special protein analyzer (Siemens BNII, Munich, Germany). The peripheral blood cell count was measured using an automatic hematology analyzer (XE2100, Sysmex, Japanes). Peripheral venous blood (3 mL) was extracted from children in the aseptic state and then placed into an ethylenediaminetetraacetic acid anticoagulant tube. The anticoagulant and blood were shaken gently and centrifuged at 8,000 rpm for 15 min. The stratified plasma and serum were stored in a refrigerator at −20°C for testing.

### BALF Sample Preparation

BALF samples obtained from the LRT were used for routine diagnostic procedures. Fibreoptic bronchoscopies with BAL were performed according to the American Thoracic Society guidelines via the oropharyngeal route in accordance with a standard operating procedure, during which 1 mL/kg saline with a concentration of 9 g/L was administered to the bronchial opening in the middle lobe of the right lung and the lower lobe of the upper lobe of the left lung. The lavage was fully recollected and used for bacterial culture and DNA extraction.

### DNA Extraction and 16S rDNA Sequencing

DNA extraction and high-throughput sequencing were performed by the Beijing Key Laboratory of Emerging Infectious Diseases Institute of Infectious Diseases, Beijing Ditan Hospital, Capital Medical University. DNA was extracted using a MagNA Pure LC 2.0 system and a MagNA Pure LC Total NA Isolation kit (Roche, Mannheim, Baden-Württemberg Land, Germany) in accordance with the manufacturer’s instructions and quantified using a Quant-iT PicoGreen dsDNA assay kit (Invitrogen, Eugene, Oregon, United States). Polymerase chain reaction (PCR) amplification of the V3–V4 region was performed with the following primers containing Illumina adaptor sequences and dual-index barcodes to tag each sample: 341F 5′-CCTACGGGNGGCWGCAG-3′ and 805R 5′-GACTACHVGGGTATCTAATCC-3′. The PCR reaction conditions were as follows: 95°C for 3 min, followed by 25 cycles of denaturation at 95°C for 30 s, annealing at 65°C for 30 s, extension at 72°C for 30 s, and a final extension step at 72°C for 5 min. PCR products were then cleaned using AMPure XP beads (item no. A63882; Beckman Coulter Inc., Fullerton, CA, United States). The amplicon sequencing libraries were constructed in accordance with the 16S Metagenomic Sequencing Library Preparation (Illumina, Inc., San Diego, CA, United States). Paired-end sequencing with a read length of 250 bp × 2 was performed on a MiSeq instrument (Illumina, Inc.) using a Miseq v2 reagent kit (Illumina, Inc.).

### Data Analysis

Reads were trimmed using Sickle (version 1.33) (Joshi and Fass, 2011). Sickle is a tool that uses sliding windows along with quality and length thresholds to determine when quality is sufficiently low to trim the 3’-end of reads and also determines when the quality is sufficiently high to trim the 5’-end of reads. It also discards reads based on the length threshold. Sequencing reads were processed using QIIME (version 1.9.0) (Caporaso et al., 2010), and an index of alpha diversity was calculated with QIIME based on sequence similarity at 97% (operational taxonomic units, OTU). The distance matrix obtained by Unifrac analysis was used for a variety of analytical methods, and the similarity and difference of microbial evolution in different samples were visualized through the principal coordinate analysis (PCA) of multivariate statistical methods. The random forest and correlation heat map were prepared with package in R 3.5.1. All statistical tests were double-tailed, and *P* < 0.05 was considered statistically significant.

## Results

### Patient Information

Among the 68 children aged 3 months to 14 years, the number of patients aged <1 year, 1–3 years, and 3–14 years patients were 21 (30.9%), 18 (26.5%), and 29 (42.4%), respectively. There were 33 males and 35 females. A total of 64 patients were included, of whom 27 presented with pneumonia or bronchopneumonia with respiratory allergy, 28 with pneumonia, and 9 with pneumonia with other symptoms, such as tracheal stenosis and tracheal softening. In addition, four patients in the tracheal group who had foreign body in trachea, were matched. The results of traditional culture and nucleic acid testing of bacteria, fungi, mycoplasma, viruses, and others were negative.

### Characterization of Core Microbiome in the BALF

**FIGURE 1:**
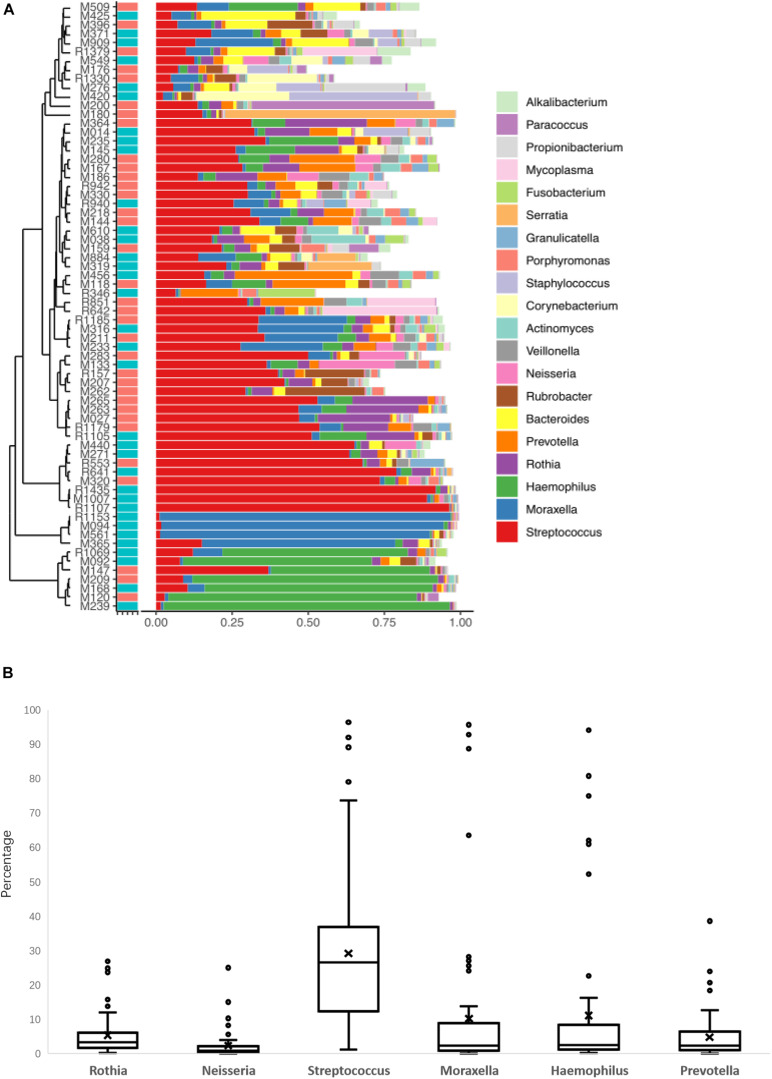
The lower respiratory tract (LRT) microbiome composition profiles for the 68 patients in this study **(A)**. The percentage of the six genera of bacteria was found in all patients **(B)**.

The LRT microbiome composition profiles were analyzed using a 16S rDNA sequencing-based method. We obtained 16 M paired-end 250 bp reads, with an overall average of ∼302,487 reads per sample. The proportion of sequence reads obtained in every genus to all genera in each BALF sample were calculated, and the top 20 genera were selected for analysis and presentation (Figure 1A). Further analysis of the core microbiome at the genus level showed that at least 6 genera could be observed in all patients and that these genera might comprise the core microbiome in the LRT. *Haemophilus*, *Moraxella*, *Streptococcus*, *Rhothia*, *Neisseria*, and *Prevotella* were detected in BALF samples from all patients, and the abundance of *Streptococcus* was the highest in this group (Figure 1B). Oropharynx and nasopharyngeal of children especially young children has a high proportion of *Streptococcus*, so the bacteria maybe enter into the LRT when fibreoptic bronchoscopies were performed. Among the 64 patients, 24 patients (37.5%) had the frequency of one genus more than 50%. In 11 patients, the relative concentration of *Streptococcus* was more than 50%, and similar results were observed for *Haemophilus* in 7 patients, *Morexella* in 4 patients, *Serratia* in 1 patient, and *Paracoccus* in 1 patient.

In these 24 patients, a TIgE level ≥ 60 IU/mL was obtained in 9 patients and a TIgE < 60 IU/mL was obtained in 15 patients. There was no significant difference in the results of clinical symptoms or other indicators between these patients and those with diverse microbiomes. The remaining 44 patients showed microbiome diversity and could be further analyzed. Because the abundance value of a single species in the sample is too high to affect the analysis of the microbiome profile, which happened in 24 samples of the 68 cases in this study, the distribution and diversity of the microbiome were analyzed in the samples of 44 cases. BALF collected from children with foreign bodies in the bronchus was as a negative control, since the microbiomes in these samples were similar to those in the normal LRT.

### LRT Microbiome in Children With AS

**FIGURE 2:**
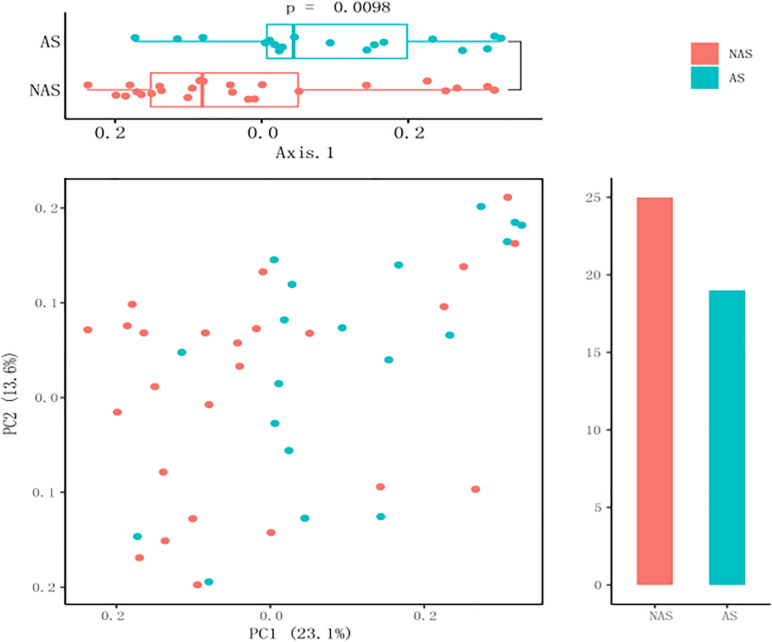
A principal coordinate analysis (PCA) plot based on 16S rDNA sequencing of 64 lower respiratory tract (LRT) samples. The scatter plot shows principal coordinate 1 (PC1) vs. principal coordinate 2 (PC2). The percentages shown are percentages of variation explained by the components. The PCA profile of microbial diversity across all samples using the Bray–Curtis distance.

Because the frequency of one genus in some LRT samples was more than 50%, and this result could not show the diversity of the microbiome, these samples were excluded from the data analysis. All the samples were collected from children with pneumonia, so we thought that the dysbacteriosis in LRT samples may have been caused by bacterial infection. Therefore, only 44 samples with relatively rich microbiome structures were analyzed. In the AS group with allergic disease (19 cases), the number of patients with asthma was 6, with rhinitis was 8, and with asthma and rhinitis was 5. In order to determine whether the children with AS displayed a different pattern of microbiome in LRT relative to the children without AS, we compared the microbiome profile of children in the AS group and the NAS group. Using a PCA with Bray–Curtis distances, the distribution of the LRT microbiome in the children of the AS group was significantly different from that in the NAS group (*p* < 0.01). This result indicates that the patients with AS have a specific microbiome pattern in their LRT samples (Figure 2).

**FIGURE 3:**
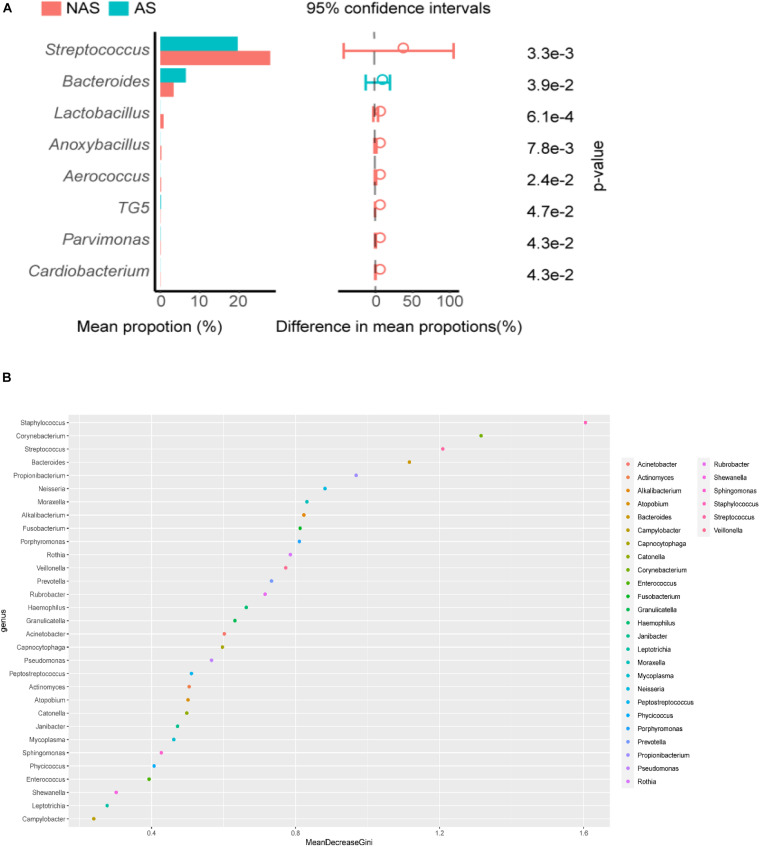
*Streptococcus*, *Bacterodies*, *Lactobacillus*, *Anoxybacillus*, *Aerococcus*, TG5, *Pavimonas*, and *Cardiobacterium* showed significant differences (*p* < 0.05) between the no allergy sensitivity (NAS) group and allergy sensitivity (AS) group **(A)**. Color spots of the relative abundances of 30 species selected for the random forest (RF) model used to distinguish samples in no allergy sensitivity (NAS) group and allergy sensitivity (AS) group **(B)**.

**FIGURE 4:**
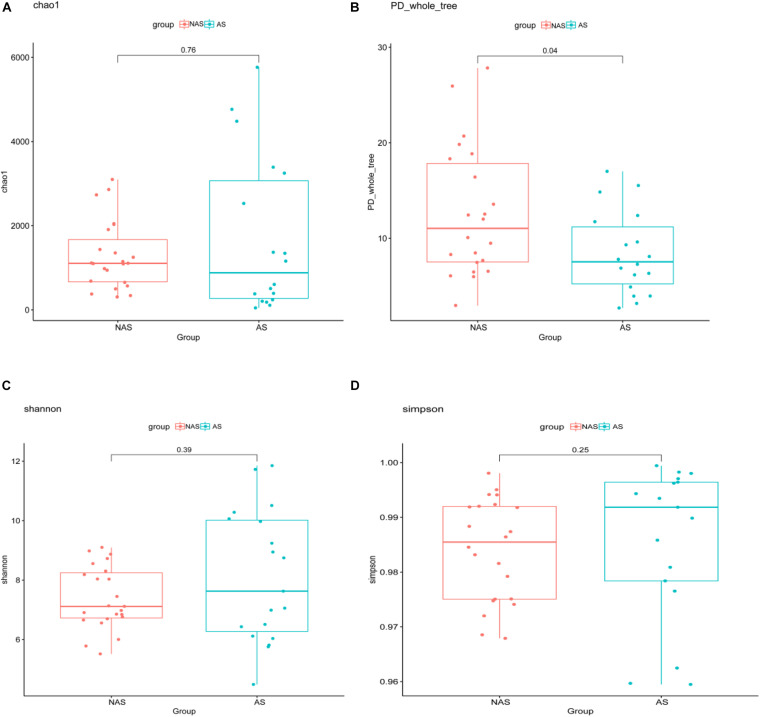
The alpha-diversity box and whisker plot of taxa richness in samples were analyzed by partial 16S rDNA sequencing. Index of Chao 1 **(A)**, PD_whole_tree **(B)**, Shannon **(C)**, and Simpson **(D)** was calculated respectively.

To clarify which specific bacteria caused the different patterns between groups, we examined the difference in the relative abundance of the bacteria between the cohorts at the genus level. Compared to the control NAS group using mean propotion caculation, the LRT microbiome in the children of the AS group had lower levels of *Streptococcus*, *Lactobacillus*, *Anoxybacillus*, *Aerococcus*, *Pavimonas*, *Cardiobacterium* and TG5, but higher levels of *Bacteroides* (Figure 3A). Futhermore, *Streptococcus* and *Bacteroides* were also screened in random forest model (Figure 3B), although *Staphylococcus* and *Corynebacterium* showed the more important role between the AS group and NAS group. This result indicated that children in the AS group have a specific microbiome pattern in their BALF samples. We assessed the genetic diversity of 16S rDNA of the microbiome in the two groups to compare alpha-diversity, including index of Chao1, Simpson, Shannon, and phylogenetic diversity (PD_whole_tree). The results showed that the index value of PD_whole_tree of the NAS group was significantly higher than that of the AS group (*p* < 0.05), indicating that the genetic diversity of the bacteria in the NAS group was significantly higher than that in the AS group (Figure 4).

### Relation of Clinical Indices and Microbiome in the Children of Different Groups

**FIGURE 5:**
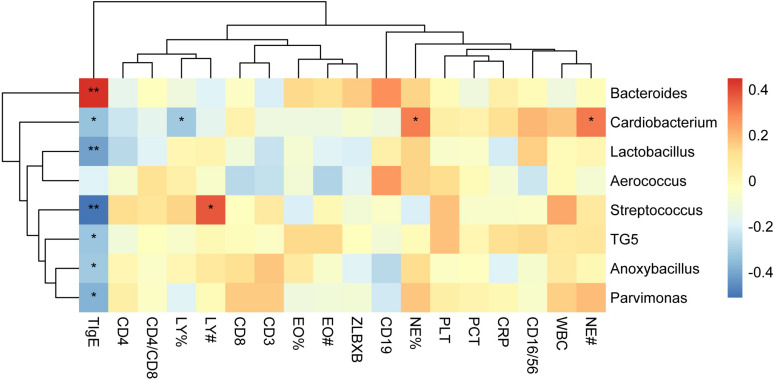
The correlation heat map shows correlations between clinical indices and the relative abundance of genera. Correlation coefficients with the color value bar are shown in the blocks. NE%, LY%, and EO% present the percentage of neutrophile granulocyte, lymphocyte, and eosinophils count, respectively. NE#, LY#, and EO# present the absolute neutrophile granulocyte, lymphocyte, and eosinophils count, respectively. **P < 0.01, *P < 0.05.

To determine the clinical characteristics of children with NAS and AS, we listed the clinical indices for the children of the two groups and compared the differences between them (Table 1). The value of TIgE, the percentage eosinophils count (EO%), the absolute eosinophils count (EO#), and the CD16/56 level were significantly different in the two groups (*p* < 0.05). The analysis of correlation between the clinical indices and microbiome which had significant different abundance in NAS and AS groups, showed TIgE was positively correlated with *Bacteroidetes* and negatively correlated with *Streptococcus* (*p* < 0.01). In addition, absolute lymphocyte count showed a relationship with *Streptococcus*, and the absolute neutrophil count or percentage of neutrophils showed a relationship with *Cardiobacterium* (Spearman correlation index | R| ≥ 0.30 and *p* < 0.05), as showed in Figure 5.

**TABLE 1:** The biomarkers in NAS and AS groups and the difference between the two groups.

	**NAS (*n* = 25)**	**AS (*n* = 19)**	***P*-value**
Age, years	3.5 (3 M–11Y)	4.3 (10M–13Y)	–
Gender (M/F)	11/14	10/9	–
TIgE (IU/mL)	25.42 (3.66–58.9)	259.24 (61–1,947)	0.001*
WBC	9.28 (3.44–19.14)	10.2 (5.07–17.88)	0.811
N%	0.51 (0.10–1.21)	0.43 (0.26–0.79)	0.087
L%	0.43 (0.13–0.81)	0.47 (0.157–0.679)	0.553
EO%	0.02 (0–0.091)	0.03 (0.01–0.079)	0.016*
N#	5.39 (1.21–23.6)	4.31 (2.2–13.85)	0.859
L#	3.80 (0.69–6.78)	4.71 (2.08–12.14)	0.380
EO#	0.16 (0–0.83)	0.31 (0.03–0.95)	0.005*
PCT (ng/ml)	0.31 (0.05–1.42)	0.24 (0.05–1.03)	0.652
PLT	397.84 (181–756)	428.78 (175–672)	0.879
CRP	15.77 (1–103)	5.97 (1–31)	0.259
CD4	36.04 (17–55)	37.12 (19–45)	0.447
CD8	26.83 (16–37)	26.31 (18–37)	0.393
CD4/CD8 (%)	1.42 (0.9–2.91)	1.48 (0.57–2.21)	0.2
CD3	68.12 (39–82)	67.31 (55–73)	0.419
CD19	20.13 (10–37)	20.13 (10–37)	0.666
CD16/56	11.41 (4–22)	10.13 (1–19)	0.012*
ZLBXB	97.33 (95–99	97.56 (95–99)	0.904

*indicates the difference between NAS and AS group, *p* < 0.05.

## Discussion

There have been many reports on the correlation between decreased diversity of the intestinal microbiome in infants and in school-age children and the increased risk of allergic diseases, such as asthma and atopic eczema, as well as the therapeutic strategies aimed at modulating the microbiome (Bisgaard et al., 2011; Abrahamsson et al., 2012; Pisi et al., 2017). The abundance of *Lachnospira* and *Clostridium neonatale*, and the decrease of *Veillonella*, *Faecalibacterium*, and *Rothia* in children at risk of asthma that these specific gut bacteria play a role in protecting or promoting development. Recently, Chiu et al. (2019) found that the interaction between specific species of intestinal microbial dysbacteriosis and IgE-mediated allergen response might lead to early susceptibility to allergic rhinitis and asthma in children. The total serum level thus forms the basis for atopic quality.

However, does the respiratory tract microbiome increase the risk of allergic respiratory disease by regulating IgE levels? In previous studies, the relationship between bacteria in the upper respiratory tract and allergic respiratory diseases, especially asthma, has been reported (Marri et al., 2013). When the nasopharyngeal microbiome of children was assessed, the virus and bacteria species that caused acute respiratory tract infection were captured. *Haemophilus*, *Morakot*, *Bacterias*, *Staphylococcus*, *Streptococcus*, ectopic *Clostridium*, and *Corynebacterium* were found in the nasopharyngeal sample of infants during the first year after birth. These bacteria not only cause inflammatory reactions (such as fever) in the respiratory tract but also directly or indirectly promote persistent asthma (Teo et al., 2015). Similar results were also reported for pharyngeal colonization of *Haemophilus*, *Streptococcus*, and *Moraxella*, which increased the risk of acute asthma or aggravated asthma in children (Bisgaard et al., 2007). In addition, *Streptococcus pneumoniae*, *Staphylococcus aureus*, *Moraxella catarrhalis*, *Pseudomonas aeruginosa*, and *Haemophilus influenzae* were cultured from the sputum of patients with asthma (Wood et al., 2010). These bacteria are also cultured during phases of exacerbations and clinical plateaus in asthma patients. Furthermore, the sputum microbiome of patients with severe asthma were different from those of healthy individuals and patients with mild asthma, and the level of *Streptococcus eosinophilus* was especially different (Zhang et al., 2012). The pathophysiological mechanisms by which these organisms cause asthma are not well known (Zhang et al., 2016). Since the nasopharynx is thought to be a microbial reservoir associated with acute respiratory infections, its microbial composition is primarily similar to that of the upper airway. There are many studies on the microbiome of the upper respiratory tract in children, but few studies have investigated the microbiome characteristics of the LRT due to the difficulty in obtaining BALF samples from children. Comparing nasopharyngeal samples with BALF samples, it was found that species abundance and diversity of the microbiome in BALF were more abundant than those identified using nasopharynx samples. *Actinobacteria* species are more abundant in the nasopharynx, while *Bacteroidetes* is more abundant in the BALF. There are differences in the levels of all species, except for *Streptococcus* (Kloepfer et al., 2018).

In this study, although the microbiome diversity in 35% of patients (24/68) was disordered, the remaining 65% of patients (44/68) showed the potential effect of the microbiome in the LRT on allergic respiratory diseases in children by analyzing the correlation between the pulmonary microbiome and serum IgE in children undergoing bronchoscopy. Analysis results with PCA showed that there was a statistically significant difference (*p* < 0.01) between the AS group and the NAS group, suggesting that the LRT microbiome was correlated with TIgE level. Compared with the AS group, *Streptococcus, Lactobacillus*, and *Anoxybacillus* were more abundant in the LRT microbiome of the NAS group, with statistically significant differences, while *Bacteroidetes* was significantly higher in the AS group, which demonstrated that enrichment of *Streptococcus, Lactobacillus*, and *Anoxybacillus* in the LRT group may suppress allergy and *Bacteroidetes-*induced allergy. Previous reports have shown that respiratory infection or treatment with *Streptococcus pneumoniae* attenuates allergic immune responses and suppresses allergic airway disease by inducing regulatory T cells (Thorburn and Hansbro, 2010; Preston et al., 2011). Furthermore, *S. pneumoniae* vaccination of asthmatic children and elderly patients reduced the number and severity of asthmatic exacerbations (Thorburn et al., 2009). In contrast, the number of *Bacteroidetes fragilis* and *Bacteroidetes intestinalis* was reported to be significantly higher in Japanese cedar pollinosis (Jis) subjects than in non-JCPsis subjects before the pollen season, and symptom scores and JCPsis-specific IgE were also positively correlated with these bacteria (Odamaki et al., 2008). LPS from *Bacteroidetes* was less immunogenic compared to *E. coli* LPS and can even inhibit innate immune signaling and endotoxin tolerance by *E. coli* LPS. So, the predominant colonization of LPS-silencing bacteria may affect certain aspects of immune education (Vatanen et al., 2016). Although *Corynebacterium mastitidis*, *Staphylococcus aureus*, and *Corynebacterium bovis* sequentially emerged during the onset of eczematous dermatitis (Kobayashi et al., 2015), especially *Staphylococcus aureus* were reported to be mainly associated with pediatric atopic dermatitis (Byrd et al., 2017), there were less reports about allergic respiratory tract diseases. In addition, diversity and decrease in the community composition of the microbiome were observed in the lower airway, which is associated with inflammatory phenotypes (Figure 1). The similar results were obtained by Bao et al., who collected BALF from protracted bacterial bronchitis (PBB) infants and tracheomalacia (TM) infants younger than 3 years old, then found microbiome diversity was significantly lower in PBB group than it in TM group (Bao et al., 2018). Recurrent cycles of infection-related inflammation of the LRT drive the pathogenesis of persistent wheezing in children.

All children were classified into NAS and AS groups according to the value of TIgE. Comparing the clinical indices of the two groups, the percentage eosinophils count (EO%), the absolute eosinophils count (EO#), and the CD16/56 level were significantly different in the two groups, which was consistent with the meaning of these clinical indices, because EO%, EO#, and CD16/56 were all reported the meaningful indicators for allergy disease (Kelly, 2018; Kelly and Khan, 2008; Fulkerson and Rothenberg, 2013). IgE production is influenced by the microbiome as germ-free mice or mice with a low diversity microbiome developed elevated levels of serum IgE early in life (Cahenzli et al., 2013). Here, the microbiome, such as *Bacteroidetes* and *Streptococcus* and, showed positively or negatively correlated with TIgE. Therefore, the respiratory microbiome, as intestinal microbiome, may also be a fertile target for the prevention or management of diseases such as allergic asthma, which are characterized by adaptive immune dysfunction (Fujimura and Lynch, 2015). Using a model of respiratory dysbacteriosis in mice, it was found that respiratory tract microbiome imbalance improved the progress of allergic respiratory diseases, and this was by promoting the local production of IL-33. This information will help to further explore the pathophysiological mechanisms of allergic respiratory diseases and provide new ideas for the diagnosis and treatment of diseases (Gollwitzer et al., 2014). IgE is most common immunoglobulin in atopic disease and plays an important role in mast cell degranulation and in initiating the T helper 2 (Th2) response. Asthma and atopic disease are typically associated with adaptive immunity with the overexpression of Th2. This is still a relatively new field of research. Although microbiome, such as *Streptococcus* and *Bacteroidetes*, are corelated with allergic sensitivity, the mechanism of the correlation between the microbiome of the LRT and early respiratory allergic diseases in children is not clear and needs to be studied further.

## Conclusion

A disorder in the composition of the microbiome was shown in BALFs obtained from children with inflammatory phenotypes. The microbiome of the LRT of children was related to early respiratory allergic diseases. As with the microbiome in the intestinal tract, a decrease in microbial diversity and a change in population composition was found lead to an increase in allergic symptoms in children.

## Data Availability Statement

The datasets presented in this study can be found in online repositories. The names of the repository/repositories and accession number(s) can be found below: https://bigd.big.ac.cn/gsa/browse/CRA003290, Project ID PRJCA003568.

## Ethics Statement

The studies involving human participants were reviewed and approved by the medical ethics committee of the Capital Institute of Pediatrics, Beijing, China. Written informed consent to participate in this study was provided by the participants’ legal guardian/next of kin.

## Author Contributions

JC and YZ wrote the manuscript, processed the sequencing data, and performed the analyses. HZ and XS performed the sample curation and sample collection. ZC and QZ curated the samples, managed the metadata. CY and GX extracted the DNA and sequenced the microbiome samples. SL and YF performed the clinical experiment. HL performed the analyses and assisted in the manuscript writing. CC and XX conceived of the project and reviewed the manuscript. JY guided the analysis and manuscript writing. All authors read and approved the final manuscript.

## Conflict of Interest

The authors declare that the research was conducted in the absence of any commercial or financial relationships that could be construed as a potential conflict of interest. The handling editor declared a shared affiliation with the authors YZ, ZC, QZ at the time of the review.
